# Creative pedagogic methods for self-guided embodied thinking and learning about animal research

**DOI:** 10.12688/wellcomeopenres.24930.1

**Published:** 2025-10-24

**Authors:** Emma Roe, Sarah J. Bailey, Louise Mackenzie, Jordi L. Tremoleda

**Affiliations:** 1 University of Southampton School of Geography and Environmental Science, Southampton, England, SO17 1BJ, UK; 2 University of Bath Department of Life Sciences, Bath, England, BA2 8AY, UK; 3 University of Dundee Duncan of Jordanstone College of Art and Design, Dundee, Scotland, DD1 4HN, UK; 4 Queen Mary University of London, Blizard Institute, London, England, E1 2AT, UK

**Keywords:** Animal research; curiosity; creative methods, Concordat on Openness; public engagement; embodied learning; arts

## Abstract

A prevailing view suggests that with greater knowledge, the public might respond differently to opinion surveys. This paper examines animal research – a domain marked by ethical complexity - to explore how curiosity and creativity can be harnessed to engage people with conflicting values and emotions. Rather than focusing solely on knowledge acquisition, the paper conceptualizes and illustrates how embodied creative methods can enable the public and industry professionals to make, to co-create their own knowledges about animal research. Three critical observations of contemporary engagement efforts are offered. Firstly, while existing approaches have made notable strides in informing and educating the public, they rely on the active participation of self-selecting learners. Secondly, engagement efforts tend to emphasise defending the principles of animal research, often overlooking the diverse everyday practices that constitutes the industry. Thirdly, a didactic, mentalist communication style prevails, reinforcing a hierarchical dynamic between expert and non-expert participants. Embodied creative methods are proposed not as tools for data collection, but as means to explore experiences and knowledge-making practices in novel ways. The paper presents three examples of such engagements authored by an artist, a pharmacologist and a veterinarian including making artwork, and two workshop activities - The Mouse Exchange and Care-full stories. These initiatives sensitively surface the complexities of the human-animal relationship at the heart of animal research. The examples show how engagements centered on creative embodied methods foster curiosity and help overcome anxieties that inhibit engagement. Reflecting on these contributions prompts a consideration about whether such activities are adequately recognised within the current Concordat on Openness in Animal Research. The paper concludes by proposing a rewording to reflect a more evolved vision of openness in animal research for the decades ahead.

## Introduction

Misinformation or a lack of information about animal research is perceived by the animal research industry as a risk that can undermine the communication of the benefits to society from, and their support for, carrying out research using animals as experimental subjects (UAR 2024–2028 Strategy). A prevailing view is that if the public were better informed and could rationally explain, perhaps they would think, feel and respond differently to public opinion surveys about animal research. For the maintenance of the democratic accountability of the sciences to the public this is an ongoing process. This paper focuses on the opportunities available for the public and those working within the industry to not *gain knowledge* but to *make knowledges* about animal research and why this approach should be taken seriously.

We begin by reflecting upon the intended audience for these communications. McGlacken and Hobson-West (2022) argue that a characterization of ‘the public‘ ( McGlacken & Hobson-West, 2022) places limitations on how informing and educating about animal research is approached. This is in part how public opinion polls on animal research work; their design constrains, and limits visions of what publics can and do care about ( *ibid*), for example by evoking imaginations of ‘societal sentience’ ( Hobson-West & Davies, 2018). Societal sentience is the notion that there is a public with clear views about the use of sentient animals. In turn, this notion of publics identified in public opinion polls becomes the imagined audience for industry-led engagement activities; the imagined public provides a justification to stakeholders when selecting styles of engagement ( Gorman & Davies, 2019). Are there opportunities to be more adventurous about the sites for learning about animal research and the style of learning by understanding the audience, or rather the learners, differently?

While rational reasoning, to explain *how* and *why* something happens, is central to science’s aims for universal and repeatable findings, it should not be the sole focus of communication about animal research. Overreliance on this approach often fuels polarized debates that limits the content of animal research communications, encouraging entrenched positions rather than airing complex reflections and sentiments that can build towards nuanced understanding. Broadly speaking this method is incapable of coping with the situatedness of heterogeneous cultures and beliefs that explain the confusing and complex diversity of sentiments. When methods like public opinion surveys are used these create gross simplifications about societal beliefs and feelings about animal research. Animal research operates within a field of ethical complexity; this comes across in the contradictory values that the public can hold towards different reasons for animal research, the use of different species, and different attitudes towards and from people who work within, with, or benefit from, the industry. Consequently, both sophisticated and innovative social scientific methods are necessary to attend to this complexity of multiple identities operating to work through what is known, felt and believed about animal research. In contrast results from public opinion polls airbrush over the complexity of beliefs and attitudes surrounding animal ethics. Methods are required that operate in two-ways to both grapple with gathering data on the complexity of understanding and feelings, whilst also tackling how to distribute insights or create space for people to encounter animal research in such a way that they can be active participants in their own learning experience on this sensitive and complex topic. By working with participants active in their own learning, the heterogeneity of publics is welcomed, but in so doing engagement activities and the transfer of knowledges and understanding about animal research between industry, regulator and publics, must become pluralized, embracing the messy, complexity of the social ( Law, 2004).

Lay stakeholders of biotechnological research using animals wear various hats and often at the same time, for example, a patient, an animal advocate, a pet owner, a sibling or parent to someone who works in an animal facility. It is often argued that where participation in democratic accountability of science by lay stakeholders is weak, imagination and willingness are required to kindle greater public and internal industry engagement on animal research, in acknowledgement of its role in the democratizing of the processes and practices of science ( Jackson *et al.*, 2005). In the current decade there are signs of greater political and scientific momentum for the innovations and opportunity to strategically align towards a shift to replacing animals through non-animal methods in research, with the hunt for suitable alternatives to animals ( Main, 2023). The current UK government has committed to the long-term goal of phasing out animal research and is working with funders to accelerate the use of non-animal approaches. Again, publics need to be part of this conversation to support and encourage the scientific infrastructural shift to non-animal methods, where possible: yet how to do it? The paper contributes to these debates by identifying epistemic characteristics and context that has informed the last decade of opening-up communication about animal research encouraged by the Concordat on Openness ( Hobson & Hoeflinger, 2024). It then moves on to share insights about where opportunities lie for working with epistemologies that combine embodied pedagogic practices with creative methods for engaging audiences differently in animal research openness. These are illustrated through some examples that have been penned by two industry pioneers and a creative practitioner, each is experimenting with embodied creativity as method or artistic practice, in the animal research space. Observations of these examples raises the question about whether these activities are adequately identifiable by the current Concordat and the concluding discussion considers whether its rewording could reflect the ongoing evolution of the vision of what could and should guide the process of animal research openness in the coming decades.

### Evaluating accounts of social relations to animal research present in current Openness Activities

In 2025 there were 132 UK institutions where animal research takes place that made commitments to being more open about their use of animals in research by becoming a Concordat signatory. Institutional signatories of Understanding Animal Research’s Concordat on Openness ( Understanding Animal Research, 2014) agree to four commitments. The Concordat’s commitments are:

- to be clear about when and why animals are used;
- to enhance communication about research;
- to be proactive in identifying opportunities for the public to find out more;
- and to report on progress and experiences of meeting these commitments.

Signatory institutions undertake activities to evidence their efforts to fulfil the four commitments. These activities have included: a website with transparent details about the number and kinds of animals used (e.g. University of Cambridge); virtual tours of animal testing facilities; education/news outputs that entwine the findings of research with the story of the animals used to develop the scientific research findings (e.g. Reading University and llamas 2022, or Cambridge University and curing paralyzed dogs, 2012); a day in the life of an animal technician film (e.g. University of Manchester 2022) and most recently an online campaign to challenge myths about animal research (Understanding Animal Research 2024). These examples illustrate valuable efforts to share more about research using animals, and to explain how animals used in research are cared for. When one looks at many of these activities the imagined public of the public opinion survey is often evident, rather than the complex picture of society with their various impediments or degrees of willingness to engage with animal research. Who is the audience seeking out these offerings of openness; and should there be more effort to go beyond conventional engagement methods? There is also a question about how the location, the site, of these engagement activities, - often online on social media and websites - , is suitable as a space to learn and become informed about animal research. And finally, with the controversy about animal research being the deliberate harming of animals and concerns for the experience of research animals, there is an absence of direct engagement with emotions, including the strong and uneasy feelings that sensitivity to these activities can stir. For, whilst anti-vivisectionist materials have a controversial history of disseminating strong, disturbing images of research animals, to afford a visceral reaction of disgust, horror and shock through how they connect to the viewer’s somatic sensibilities ( Greenhough & Roe, 2011), where are the opportunities for the research industry to work with these same sensibilities but to cultivate other ways of learning through feeling? Somatic sensibilities convey the human ability to relate with and through their body to the embodied experience of another body, in this case the living animal. Altogether, there is a place for the social sciences and humanities to work in partnership with professional artistic practitioners who can bring their skills around the bodily dimensions of social relations to explore how encounters with animal research could be curated differently.

Davies *et al.* (2024a) argue that the social sciences and the humanities have skills and insights that can support understanding the social relations around animal research. Andrew Sayer (2011) argues that social science whilst making a valuable contribution through explaining and understanding phenomenon – more akin to the role of Science - must also evaluate how fairly or adequately social life and its relations are accounted for. By bringing a social science perspective to the challenges around communicating about animal research the intention is to address Sayer’s second point, to evaluate how social relations to animal research are accounted for through current dominant approaches and activities for promoting openness about animal research. What ways of relating to animal research appear absent and unaccounted for? Could the introduction of embodied methods to engage publics, that work with somatic sensibilities towards research animals, address current oversights and, or anxieties that limit much contemporary animal research engagement activity?

The first observation is that existing approaches to these activities whilst heralding significant achievements in informing and educating on animal research, predominantly do this through requiring an audience to actively show-up to learn about animal research. There is an expectation of an audience that will seek-out openness activities, like data release events, open days, website improvements; or join-up the dots about medical research advance using animals and why animals are needed in research. Notably, despite the everyday receipt of medical treatment as informed by animal research, this connection is never explicit (cf McGlacken & Hobson-West, 2024). Contrastingly, the language of animal testing and cruelty-free is associated with shopping for beauty products, through the effective use of the declared absence of ‘animal testing’ within the branding of beauty and cosmetics chain ‘The Body Shop’, and others. Interestingly, the phrase ‘animal testing’ therefore is a term more familiar to the public than the term ‘animal research’, and they refer to subtle but important differences to the context for animal use. To test can be a regulatory requirement, whereas to research is to investigate and develop new knowledges and understanding with the distant blessing of a peer-review community. And whilst it is argued that humans adopt the coping strategy of cognitive dissonance ( Engel *et al.*, 2020) frequently referenced in relation to the eating of animals, and which certainly holds relevance to responses to animal research, at another level there remains a democratic society expectation for a connection, interest and awareness to be fostered.

The second observation is activities predominantly focus on defending the principles behind the use of animals in research, such as the value of the scientific findings that comes from the animal experimentation. These activities avoid confronting and acknowledging the emotional reluctance to engage with animal research from those members of the public who experienced anti-vivisectionist materials and switched-off ( Jasper & Nelkin, 1992; Turner, 1998); and that it is normal to have uneasy feelings about the norms around animal research, and that those working in the industry experience those feelings too. Institutional efforts often shy away from direct engagement on the topic of ethical concern, that is details on the processes and practices of everyday scientific knowledge-production, instead prioritizing engagement with messages about its societal value. To generalize the emphasis is on providing evidence for maintaining the status quo of the ethical principles via short-hand philosophical arguments for using animals in research, with reassurances about the regulatory codes that scientists operate within and the attention to animal welfare.

A third observation is the predominance of a mentalist pedagogic approach ( Macedonia, 2019). Within this learning practice, the conventional didactic and mentalist style of communication instills a knowledge-hierarchy between the expert teacher and the in-expert learner, and the content is delivered through abstract facts and figures. This approach whilst of value in compiling abstract arguments is packaged and presented in a way that precludes opportunities for acknowledging felt, emotional responses and connection to the lived experiences of receiving life-changing medicine, or caring for and thereby having close affectionate relations with (pet) animals. There are increasing arguments for creating a learning environment that can properly account for the vastly various experiences of the learner that in turn shapes a learners’ curiosity and how knowledges can be received and felt, explored and articulated: one example of this type of work in relation to animal research is the Mouse Exchange ( Roe *et al.*, 2024b). Relatedly, Roe & Greenhough (2023) and in Anderson *et al.* (2023) have argued that what Berlant terms ‘affective realism’ is employed by animal technicians who care for research animals, enabling them ‘to continue in their working role, providing deep attentive care for the individual (in this case animal), or even the collective, whilst also experiencing the animals’ pain, loss, suffering and sacrifice as personal harm’ ( *ibid*: 155). The register of affective realism, this human quality identified by Berlant and witnessed in practice with research animals, could be a way into engaging and relating to publics to bring in quite different sites, situations and materials for engagement and openness. It’s pedagogic starting point is an alternative to the rational, mental, cognitive practices of detachment that predominates the field of animal research ‘openness’ activities.

Social science research methods have been innovating to study and research ‘affective realism’ and the diversity of embodied experiences. This has led to an upsurge in interest in the opportunities around creativity through working with creative practitioners. In this paper we take what has been learnt in the social sciences and humanities of how to engage creative methods in engagement activities that draw upon the affective, embodied experiences of humans. We show how creative methods can deftly negotiate the visceral discomfort many feel about animal research which can deter its engagement, and also can challenge the widespread belief that knowledge accumulation will improve public opinion towards animal research. The examples in this paper show what is achievable through creative embodied methods, where learners are perceived as experts in their own learning journey, guided by their own curiosity and participation level. Such methods use the idea of “taking materials to participants and seeing what they build and what questions they ask, rather than offering them an existing vision of animal research about which to ask questions” ( Roe *et al.*, 2024a: 423).

In the paper’s conclusion we outline opportunities to take this approach to empower wider publics to develop deeper and more nuanced understanding of animal research, and that this could be facilitated by revising the Concordat Commitments. Should communication with media and the public be *diversified*, rather than *enhanced*? Is clarity about ‘when, how and why animals are used in research’ what the public wants to know, or what those in the industry feel most comfortable to discuss? Can employing imagination bring a different range of opportunities for the public to follow their own curiosity about animal research? At the heart of these questions is a belief that through curiosity publics can become ‘capable of comprehending and accepting [animal research’s] complex and often contradictory ethical challenges’.

## Introducing creative embodied methods

The progress of the social sciences to embrace what creative arts practice can bring to its own practice, as something called ‘creative methods’, can be traced variously. Across the social sciences the use of creative methods ( von Benzon *et al.*, 2021) which draw on disciplinary expertise traditionally associated with the humanities and the arts ( Bolt & Barrett, 2019); has grown. Social science has traditionally used talk-based methods to understand what society thinks about things such as interviews, focus-groups, questionnaires ( Davies & Dwyer, 2008), with a focus on gathering opinions and attitudes. However, interest both in human behavior, and in how cultures of emotion and feeling shape the process of learning, reacting and talking about something, has left the social sciences looking for novel methods to sensitively engage in what talk alone cannot seek to understand. Participatory action research ( Kindon *et al.*, 2007) has formed a central part of the energy around the rising relevance and potential of creative methods, as they have pushed forward an ontology ‘that suggests that human beings are dynamic agents capable of reflectivity and self-change, and epistemology that accommodates the reflexive capacities of human beings within the research process’ ( *ibid*:13). Turning specifically to the contribution of the sociology of science and technology studies, the thinking of John Law is pertinent. In his seminal text *After Method* (2004) Law argued, social science methods which have traditionally attempted to replicate scientific methods, in doing so, have overlooked many social phenomena. As Law writes

‘Pains and pleasures, hopes and horrors, intuitions and apprehensions, losses and redemptions, mundanities and visions, angels and demons, things that slip and slide, or appear and disappear, change shape or don’t have much form at all, unpredictabilities, these are just a few of the phenomena that are hardly caught at all by social science methods’. ( Law, 2004:2)

Law’s work emphasizes a re-conceptualising of how we understand human thought and action across the social sciences, as not wholly rational, but as affected by feelings, materialities and cultural context; his thinking in many respects is echoed later by Sayer (2011). Creative methods scholar Estelle Barrett explains that ‘scientific research deals with a number of conventions that relate to materials and methods: assumptions, apparatus, instrumentation, procedures, observations, methods of data collection, ethical considerations, safeguards and calculations. In established fields of research, many of the above are relatively fixed and pertain to the scientific method’ ( Barrett, 2007: 191). They write ‘in artistic practice, we constantly question the underlying assumptions and meanings related to the materials and methods that we use – it is not just about making meanings with what we have at hand, but of making new ways of making meaning through practical invention’ ( *ibid*). In the context of social science and humanities applying creative methods, the ‘making of new ways of making meaning’ is achieved through practical activities that allow participants to question assumptions and unsettle meanings about materials and methods; the audience becomes active participants, content-makers rather than viewers. Creative facilitation can foster less-established conversation topics through using props to initiate responses and relations, helping the process of finding words and expressing feelings related to the topic of interest. The material props that are a starting-place for engagements could be in the form of sounds, smells, video content, images, fabrics, objects. The content made is new thinking, unexplored feelings, images, text, all of which could be disruptive to taken for granted assumptions.

The opportunity to develop creative activities linked to social science research findings was funded through the Wellcome Collaborative Award, within the Animal Research Nexus research programme (2017–2023). Looking back, the Animal Research Nexus (AnNex) team’s interest in developing ideas across the animal research industry about how publics and stakeholders engage with animal research, was initially inspired by conversations at a workshop in 2014 with animal research industry stakeholders. At this 2014 event research questions were formulated about ‘where opportunities lay for greater and meaningful public and stakeholder engagement in the policy and practices of animal research? ( Davies *et al.*, 2016: unpag.) And it was also asked ‘what, and in what contexts’ did different publics want to know about animal research?’ ( *ibid*). Consequentially, from the outset it was a broad aim of the AnNex research programme to change cultures of communication about animal research. This led to subsequent activities throughout the research programme to attend more closely to the process and sites of knowledge-making and sharing with stakeholders – professional, students and lay audiences of animal research. Greenhough *et al.*’s Care-full stories ( Greenhough & Mazhary, 2021) and Roe *et al.*’s Mouse Exchange ( Roe *et al.*, 2024b) are two examples from social scientists who have brought Law’s intervention about social science methods, to the reshaping and making of different experiences around knowledge-making and sharing within animal research. Greenhough and Roe have developed these creative methods from initial ethnographic and interview-based social science research with those caring for research animals ( Roe & Greenhough, 2023). To that aim when designing our closing conference ‘Researching Animal Research’ on 30 ^th^ March 2023 we wanted to include a session on ‘Creative methods in animal research’.

The ‘Creative methods in animal research’ session aim was to showcase, firstly, the work of creatives who have been in conversation with AnNex researchers because they engage through their artform with animal research, and secondly, how creative outputs from the AnNex project have been taken up by external stakeholders. To offer a sense of the richness of the discussion on the day, we are grateful to three of the five panelists who agreed to contribute a short piece based on their presentation on that day to this article. On the day, the three session panelists included creatives playwright/sociologist Maisie Tomlinson, artist Louise Mackenzie, and UK interactive theatre makers, The Lab Collective. Each have produced art-forms that engage with animal research. Maisie Tomlinson provided us with insights into her process of writing a stage play inspired by animal research, called *What a Mouse Knows*; and reflected on artistic practice on animal research that troubles relations between fact and fiction, available here. Louise Mackenzie shared her experience as an artist in performing openness around animal research and discusses that in the following section. The Lab Collective spoke about their partnership with the AnNex team in developing Vector – an interactive and immersive performance ( Crudgington *et al.*, 2024). Animal research professionals on the panel were - Prof Sarah Bailey (University of Bath, UK) and Dr Jordi Lopez-Tremoleda (Queen Mary University of London, UK). They both explained why they were drawn to using creative methods and the different types of conversations that their use had facilitated; they share their reflections in the next section.

The next section consists of three short pieces written by three of the contributors to the panel. It includes the contributions of one of the creatives –an artist whose work has explored the practice of using nonhuman life as a resource (see Mackenzie). It also includes the contributions of the vet and the pharmacologist who both identify as part of the mainstream animal research community. They speak about what they and the communities they work with, and around – pharmcology students in the first case (Bailey), and animal care staff in the second case (Tremoleda), - gain from working with creative activities designed by social science researchers of animal research. Collectively the papers illustrate opportunities through creative practice to advance animal research openness and engagement. They contribute to discussion on the value of the growing diversity of creative engagement activities within and about animal research. These examples of creative engagement speak to the complexity of the human-animal relationship at the heart of animal research, bringing into the open certain experiences and practices that rarely receive public attention. It is important to note that this is not a research method paper, the creative methods described are not in use to gather research data but to explore experiences and knowledge-making practices around animal research in a different way.

## Reflections on creative methods in Animal Research

### Performing Animal Research by Louise Mackenzie

Growing up in the UK in the 1980s, I witnessed a period of intense social engagement with animal research, where television advertisements showed graphic imagery of bloodied furs ( Li, 2019), and activism towards researchers escalated to acts of terrorism ( The Scottish Herald, 1990). Inevitably, changes in legislation followed and today, public perception of animal research is more nuanced. There is a broad social acceptance of laboratory animal research in the UK, so long as animal use and suffering are minimised and health outcomes are realized ( Davies *et al.*, 2024b). Is it the case that we have learned through bitter experience to distance ourselves from animal research in the same way that a surgeon must distance themself emotionally from the body that they will cut into on the operating table? Is abstraction a coping mechanism? And if so, is this a tacit acceptance that animal research is no longer on the table for discussion? I propose that *performing* animal research can offer a way through this.

Through my engagement with the Animal Research Nexus, I have found myself considering the role of performance in my practice and the practice of others in relation to the subject of animal research. Performance in art historical terms can be seen, ‘as a way of breaking down categories and indicating new directions’ ( Goldberg, 1984). Unlike conventional theatre, the key to performance is that ‘each time an artist does a piece, it can be new, different, and open to spontaneous change and growth’ ( Brown, 1984). The contract between performer and audience is based on the understanding that the unexpected may occur. Performance has all the potency of a chance interaction - a space for uncertainty which offers potentially limitless outcomes. Performing animal research therefore has the potential to generate much needed dialogue amongst the varying stakeholders in the field – given the right ingredients.

What might those ingredients be? Whilst not exhaustive, I will point to examples of performing animal research that could be part of the mix.

***Objects of performance***

Choice of object(s) used in performance, and how the performer guides actions around them, are key to the content and tone of the resulting dialogue. Here I consider the objects – a medicine label, a mouse and multiple narrative scenarios from three engagement activities that use creative practices developed by the Animal Research Nexus team.

The engagement activity Labelling Animal Research ( McGlacken & Hobson-West, 2024), asks participants to create labels for medicines that indicate whether a product has been tested on animals. The performative act of designing the label enacts a deliberate remoteness from the research animal. Rather, the object of interest in this participatory activity is the label itself and the information it conveys. The seemingly simple task of performing the role of label maker becomes a complex journey as participants begin to understand the emotive ethical context that surrounds how we communicate animal research in relation to medicine; and to consider how this might differ from the use of production labelling on food or beauty products that address the welfare of the farm animal or speak to the absence of the animal.

In the Mouse Exchange project ( Roe *et al.*, 2024b), participants learn about the laboratory mouse, not from the point of view of its role in experimentation, but rather its origins and how it is cared for. Drawing from performance art ( Conway & Hayhoe, 2006), participants have the opportunity for dialogue around a table whilst creating their own felt laboratory mouse, from its ‘embryonic’ state – as felt pieces and thread drawn from a cool box ‘biobank’. Worth noting is the closing stage of this activity, where participants must contend with the emotional context of leaving behind the mouse they have made, adding it to an infinity box that marks its transition into the life of a multitude of lab animals. Constructing a fictional mouse focuses the performance (and the accompanying dialogue) on another singular object, this time the animal. The presence of the mouse could initiate dialogue around how or why animals are used in research and these avenues are not closed to participants, but interestingly, the performative act of constructing the mouse and adding it to the imaginary lab directs the performance - and hence the conversation towards how the animal comes to be in the lab and how it is cared for as a part of that process. As with the medicine label, the object (in this case the mouse) guides the direction of dialogue, yet leaves plenty of space for participants to engage with the themes of the work on their own terms.

By contrast, Vector is a wholly immersive theatre experience ( Crudgington *et al.*, 2024). Participants become the decision-makers required from an Animal Welfare and Ethical Review Body (AWERB) through role-play and game design. Participants are responsible for choosing one of four different species to be used in research. They encounter facts about the relative costs and benefits of using each animal and compete with other teams in advocating for their choice. In this performance, there is no singular object to contend with, rather there are multiple narrative scenarios. Vector operates like a story-mode online game – participants feel free to make a range of choices, yet each has been carefully orchestrated to contribute to dialogue around harm-benefit analysis. Thus, the conversation associated with this type of performance is highly focused, with participants being led from one decision to the next, and where tailored information is revealed in relation to decisions made. Whilst undoubtedly raising participant awareness of ethical decision making in relation to animal welfare, the complexity of an immersive experience like Vector is more strongly guided by a ‘game creator’, resulting in participants being led by the process, in contrast to the previous examples where participants lead their process and depth of engaging in animal research. Whilst there is ample opportunity for open dialogue in Vector, the sense of ‘being pawns in a game’ may devolve participant responsibility as they try to observe (or otherwise) the rules of the game, more so than the less complex opportunities for dialogue afforded by Mouse Exchange and Labelling Animal Research.

Vector highlights a further ingredient in performing animal research – the influence of the creator. The creator’s hand is present in generating imaginary scenarios for audiences to engage with: label, mouse, AWERB. The simpler the scenario, the more opportunity the audience has for dialogue, for chance interaction, with open-ended outcomes. With a highly articulated game structure, the audience response may be specifically channeled.

In each of the above examples, the object of performance is a substitute that allows the audience to distance from the living animal at the heart of animal research. Whilst abstraction, play and speculation have a place in imagining futures for animal research, there is another form of art which brings the live subject back to the table for discussion. Process-led and event-driven art practices are a staple of new media artists working with biological materials. Artists who work with live biological material are by nature engaging directly with questions of ethics and care in the making, exhibiting and maintenance of works of art. Perhaps by widening the forum for who engages with the animal in research through art, we can document and learn from personal experiences in ways that not only focus on the animal in research but on the individual voices that work with the animal.

***Performing through practice***

Artist Kathy High engages directly with laboratory research animals, but from a perspective of compassion and care that is deeply entangled with her own life experience. In *Embracing Animal,* High examined relations of care in a species of laboratory rat bred specifically to study an illness that High has lived experience of ( High, 2004). Through direct and personal engagement with these animals, creating heightened experiential environments, routines and even homeopathic treatments for the rats, High was able to explore questions such as – how might we reframe our understanding and experience of laboratory animals? and how could their lives be sustained in ways other than currently experienced? In doing so, High highlights the complex and nuanced relationship that medical science has with animals. Her deep care and respect for these rats without whom her own illness would not be better understood is manifest in her very public celebration of their lives. I regard High’s work as an example of *performing through* animal research, as distinct from performing animal research. High’s research was undertaken with animal researchers and with the animals themselves, creating a deeply personal performative engagement that addressed the how and why of using animals for medical research, and even led to finding the animals homes, after the work ended.

Like many of my contemporaries who work with life as subject matter, I perform actions as a part of my practice to interrogate and destabilise existing structures and concepts. When I go into a laboratory, or other domain of expertise outside of the realm of art, my preferred mode of operation is to work with the tools and techniques of that specialism. I may not do this ‘correctly’. I often begin with the help and guidance of experts, but I choose to act by reading that space, its methods and behaviours through my own subject position. I suppose this is a form of ethnography, an auto-ethnography perhaps, but one in which through performing the actions of another discipline, rather than simply observing them, I am deliberately tracing my difference within it.

I have a complicated relationship with animal research. Whilst I am uncomfortable with including animals in my own art research without their consent, I have tested this boundary through an act of genetic modification. I am inherently curious about living beings and our interactions with them, particularly our inevitable, yet conflicted, use of life as a resource. In my work *Evolution of the Subject* ( Mackenzie, 2018a), I chose to explore the emotional context of the act of genetic modification. An act so technologically streamlined that it is now within the toolkit of artists and designers as well as scientists ^1, 2^ ( Davis, 1996). In this work I genetically modified *E. Coli* bacteria, often considered the lab-rat of the bacterial world given their preponderance to being experimented with. My genetic modification was for these bacteria to hold within their bodies a question (coded as DNA) from me to the organisms. What I had not anticipated was how responsible I would feel for these living bacterial beings, nor the sense of shame – and at the same time, relief – I would have when periodically terminating their bacterial lives to control their volume in the small laboratory space allocated to me. These emotions have stayed with me as I consider the importance of *performing* genetic modification and how this act helped me to grasp more fully the implications of using life as a resource. To amplify these emotions, I developed the short film *Zone of Inhibition* based on a workshop I ran in which scientists and other researchers had the opportunity to genetically modify life, before being questioned about their actions by the future evolved kin of the life that they had modified ( Mackenzie, 2018b).

The use of life as a resource is still an uncomfortable truth. As the UK Concordat on Openness on Animal Research acknowledges: ‘ *accurate communication of harms done to animals in research remains a difficult topic for the research community*’ ( Understanding Animal Research, 2017). Which leads me to ask where transparency is important - whose experiences matter to the processes of making transparency in animal research?

***Who needs to be honest, and with whom?***

When animal research is undertaken, who really cares? The question of care is so often loaded, with the weight resting disproportionately on different shoulders ( Puig de la Bellacasa, 2017). There are the technicians who tend animals in research facilities; the researchers for whom – without animals – their research could not continue; the innumerable individuals within society whose health depends upon animal research and those members of society who wish to see an end to animal research. This leads to what Roe and Greenhough have described as an ‘emotional division of labour’ ( Roe & Greenhough, 2023), separating those who bear the emotional toll of caring about or for research animals from those who bear the burden of the wider economic implications of research and the place of animals within it. Thus, the question of who needs to be honest when discussing animal research is multi-faceted. As we increasingly understand that ‘public’ is not a homogeneous term ( Mahoney, n.d.; Nerlich, n.d.), the question of how to engage in dialogue around animal research must take into account the needs of highly diverse (and at times, intersecting) stakeholders.

By teasing out some of the ingredients of performing animal research as demonstrated by Animal Research Nexus and others, I have attempted to lay the groundwork for developing an approach to creative engagement with animal research that moves beyond co-opting the arts to one that embeds performance within practice. Arts-led projects can do more than engage a general public, rather they have the capacity to bring all of the voices who need to have honest conversations, to the table. By including artists, social scientists, scientific researchers, animal technicians, vets, patient recipients, animal activists and perhaps even (at least speculatively) the animals themselves in arts-driven projects, conversations around animal research can become as multi-layered and multi-dimensional as they need to be.

### Adapting the Mouse Exchange Toolkit for pharmacology undergraduates: a student-led introduction to animal research by Sarah J. Bailey

Animal research plays an essential role in biomedical research and the development of new medicines. For undergraduates in the biomedical sciences, it is important that they have an education in animal research so that they can appreciate how and why animals are used in interpreting the scientific literature. It is also important to have an understanding of the ethical considerations and the harm-benefit analysis that underpins the humane use of animals in research. In the biopharmaceutical industries there are consistent concerns regarding the skills gap in relation to ‘physiological modelling’ and in vivo research skills which places these skills as a high priority in undergraduate pharmacology courses. The British Pharmacological Society’s (BPS) curriculum for the use of research animals outlines the knowledge, skills and attitudes that undergraduate and taught masters degree programmes should acquire. There are core learning outcomes which include legal frameworks and ethical principles, experimental design and analysis, critical evaluation skills that are built into many pharmacology and biomedical science undergraduate programmes. Alongside this, students should demonstrate a respectful and considerate attitude to research animals, an awareness of the culture of care and a commitment to animal welfare across the research process. How best can we instill these attitudes in undergraduates?

**Figure 1.**
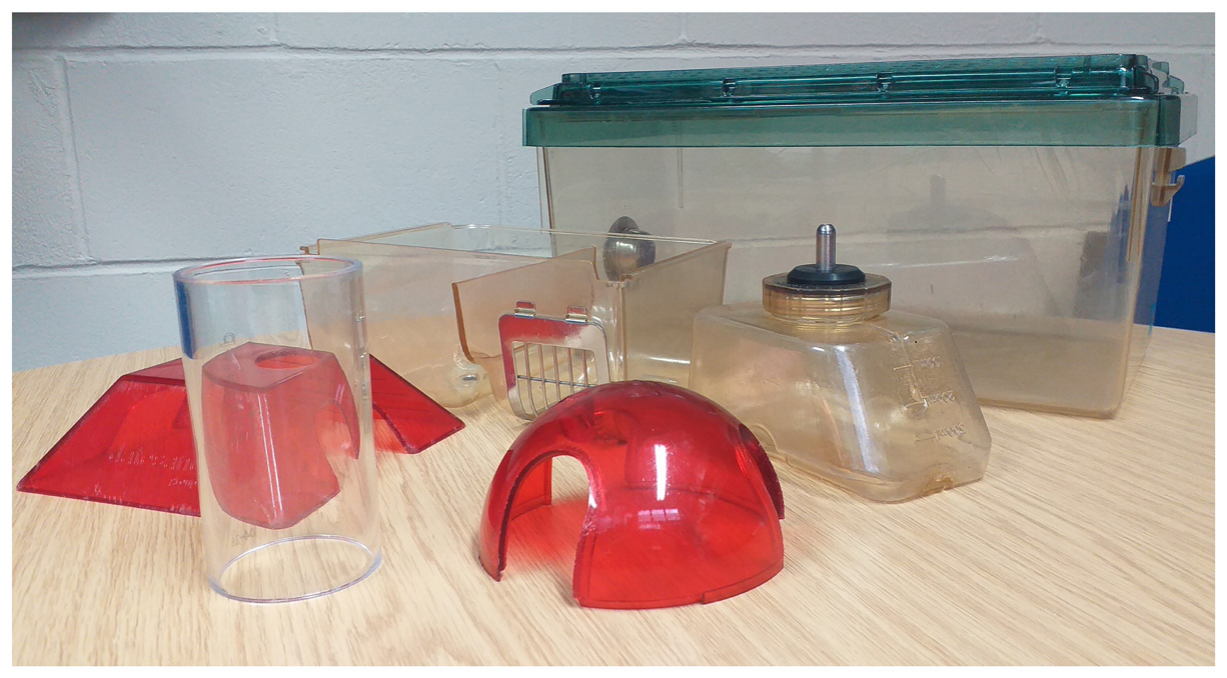
Objects used to house laboratory mice. From right to left: standard individually ventilated cage for mouse; water bottle; enrichment dome (translucent red); food hopper; enrichment fort (translucent red); mouse handling tube.

For three cohorts of first year pharmacology students (35–45 students per cohort), we have piloted a student-led approach to discussing how research animals are cared for using the Mouse Exchange Tool Kit. This public engagement activity was created as part of the Animal Research Nexus project where participants can craft a felt mouse, engage in dialogue and reflection about the complexities of creating and caring for mice in research. We adapted this tool to explore first year pharmacology undergraduate attitudes to research animals and consider the importance of a respectful and considerate attitude to research animals within the culture of care framework. In a 1h workshop, small groups (n=6-8) of students sat with a facilitator who initiated conversations that were then developed by the students. At the same time, students were invited to make a research mouse from crafting materials. A basic felt body shape was prepared in advance so students needed to use a needle and thread to make stitches to create eyes, nose/whiskers, a tail and add ears. Students also had the opportunity to explore different objects that are used to house research mice ( Figure 1). Having made the mouse, students were invited to complete a ‘mouse passport’, analagous to a ‘cage label’ that would be used in an animal facility. This activity encouraged the students to think about how their ‘research mouse’ might be cared for, what characteristics the mouse might have and what they would like to have happen to them. Qualitative comments were also captured throughout the sessions and students asked to leave feedback using post-it notes/mentimeter. Institutional ethical approval was not sought for this activity as it was a student-led education activity. Students consented to the activity by attending the class.

**Figure 2.**
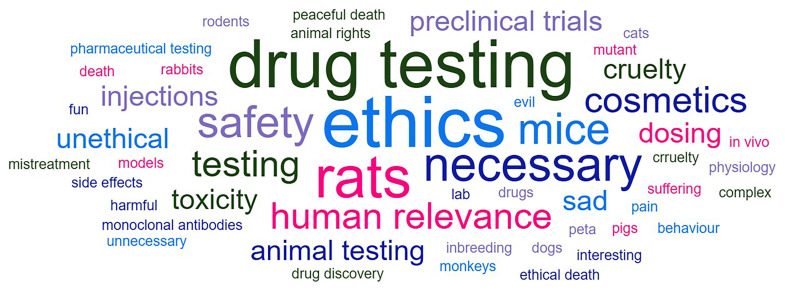
Word cloud highlighting initial student responses to the question ‘what do you think of when you hear the words ‘animal research?’ Undergraduates have no prior teaching about animal research. On arrival at the class Mentimeter poll is conducted to gather responses to this question. In this word cloud visualization, the most common responses appear larger. Responses were collected from 83 students across 3 iterations of the workshop.

**Figure 3.**
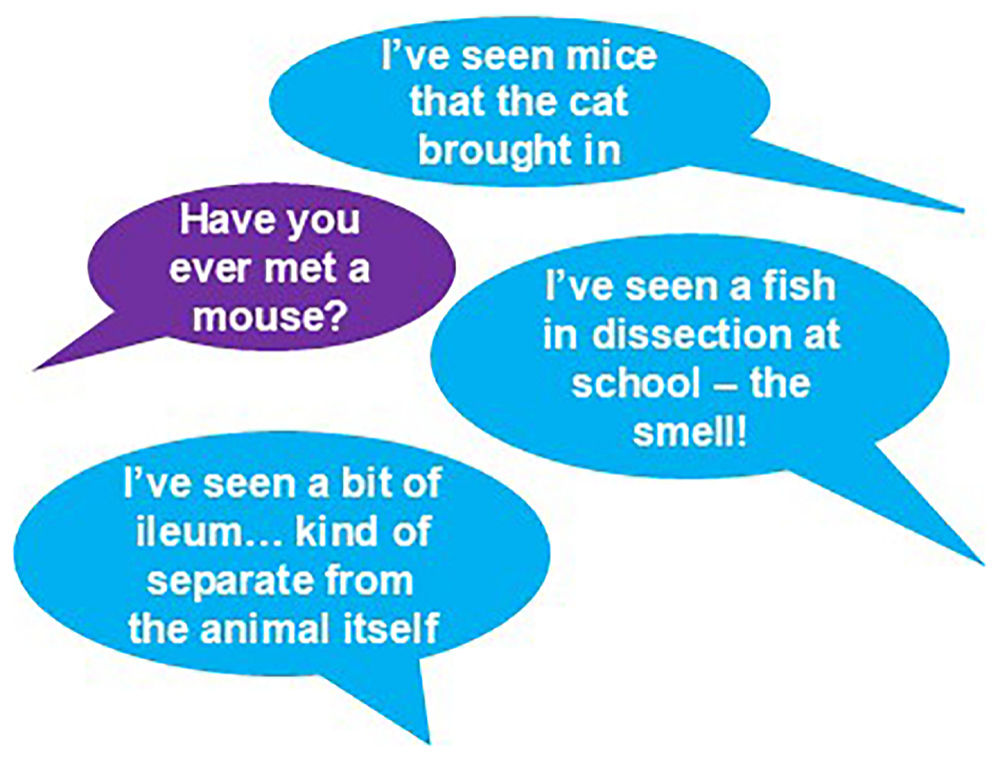
Student responses (in blue) to the facilitator asking (purple): have you ever met a mouse?

**Figure 4.**
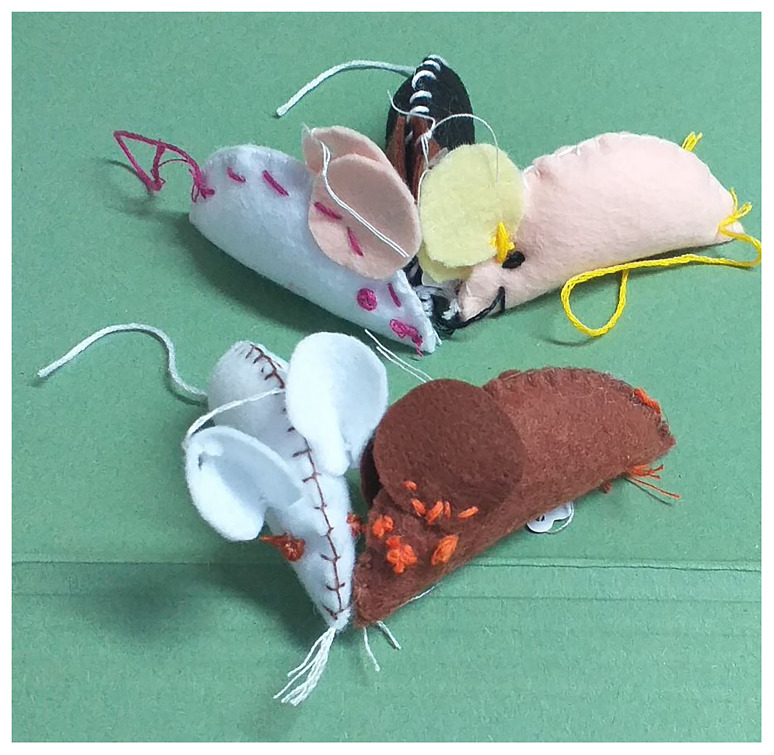
Examples of completed ‘research mice’

**Figure 5.**
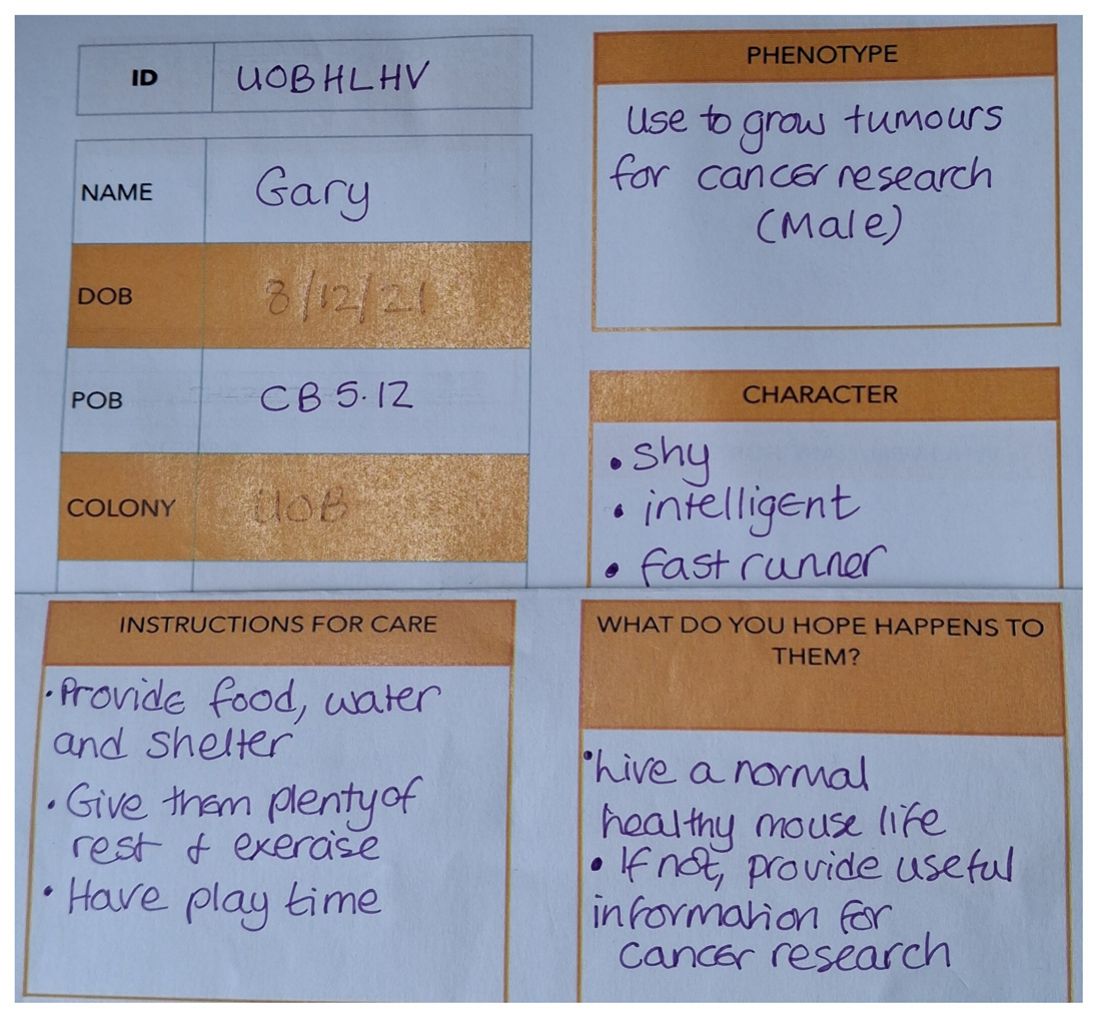
Example of a ‘mouse passport’ that each student completed for the mouse that they crafted.

On arrival in the class room a Mentimeter poll was used to gather initial thoughts on the topic of animal research ( Figure 2). Students were invited to raise any of these topics or questions with the facilitator at the table during the course of the session. Conversations were started by facilitators with a common opening question: have you ever met a mouse? ( Figure 3). Conversations then developed. Further open questions were used as prompts if required e.g. where do you think laboratory mice come from? What do you think happens to a research mouse? A challenge for the facilitators was to let students talk, or not, rather than to direct the conversation. On occasion, conversations developed between the students and diverse experiences were shared. For example, a student who came from a farming family readily accepted the idea of using mice in research. Others, with different backgrounds who had kept animals as pets felt more emotionally connected to the idea of ‘harming’ an animal by using it for research. The nature of the workshops enabled facilitators to better understand where the students were starting from. The students demonstrated limited prior knowledge of, but much interest in, where laboratory mice came from and the practices and processes of animal husbandry. Using creativity as a way to engage students and foster discussion enabled students to deepen their understanding. Posing open questions helped extend the thinking of students and allowed them to make connections to their own experience that they might not otherwise have made. Having created the mouse ( Figure 4), which is soft and tactile, the students develop a connection with their ‘research mouse’. This was evident when students were asked to leave their mice behind at the end of the activity. Students were asked to identify their mouse by making a hole in a felt ear, with a hole punch tool, and adding a small tag. Mice were then placed in a laboratory animal cage. A small number of students were evidently disappointed not to be able to take their mice home with them. Further evidence of a relationship with the ‘research mouse’ was revealed in the ‘mouse passport’ ( Figure 5). One student asked ‘Who is caring for the mouse? I can care for it…. Can I?’. When asked ‘What do you hope happens to them?’; student answers were grouped into 4 categories ‘they are used for ethical and useful research’ 46%; ‘they survive/live a long and happy life’ 26%; ‘they escape/are released’ 14%; ‘humorous e.g. sees England win the world cup’ 14%.

At the end of the workshop, students were invited to complete a post-it sticky note with ‘one thing you have learned’ in the session ( Box 1). The sticky notes indicated that around 30% of students comment on issues relating to the care and husbandry of animals. A similar number comment on learning about the humane methods of killing research animals. ‘What happens to a research animal at the end of the experiment?’ was asked by at least one student in each small group. This opened up a conversation about how research animals are humanely killed at the end of experiments and factors influencing the choice of methods. After the session, students were asked to evaluate the actual format of the workshop for delivering taught content. Students described it as ‘very engaging and informative’, ‘thought provoking’ and ‘enabled discussion of valid and important issues’. One student wrote “At the beginning of 'crafting a felt mouse' I saw the activity as a pointless exercise. However as the session progressed I felt that the activity was a good method of starting discussions between students and researchers in a setting that felt less intimidating than a standard q&a.”

**Box 1. Examples of what students say they have learned during the Mouse Exchange workshop when asked to record ‘One thing I have learned’.**

**Table:** 

How lab mice are cared for	Mice require enrichment
Some lab mice are bought in and others are bred at the University	The most ethical way to hold mice
Where animals are kept on campus	Mice are checked on a daily basis
The way mice are humanely killed	Lab mice live for about 2 years
Mice are expensive	How mce are treated during an experiment

To conclude, within higher education there is increasing emphasis on the benefits of student-led inquiry as a pedagogical approach ( Ashwin *et al.*, 2020; McCabe & O’Connor, 2014). The workshops provided a safe space for opening up conversations about care and respect for research animals in a way that other, more didactic, introductory approaches had not previously done. While the small group teaching approach delivers high quality, student-led, engaged teaching, it is resource intensive (staff, preparation of materials) and not suitable for large numbers of students. Instilling a respectful and considerate attitude to research animals and their tissues, and a knowledge of the ethical priniciples underpinning the use of research animals are core learning outcomes of the British Pharmacological Society’s curriculum for the use of research animals. This interactive and creative approach enabled students to develop the desired attitudes towards research animals and deepened their learning by making an emotional connection with their crafted felt mice. Furthermore, such creative activities have the potential to engage students in active and experiential learning supporting the learning cyle through concrete experience, motivating the students to reflective observation and abstract conceptualisation seeking how ideas can be applied to different contexts ( Kolb & Kolb, 2017). The staff facilitating the workshop also engaged in active learning, developing their understanding and perception of the factors affecting undergraduate attitudes to animal research.

### How creative facilitation of professional and emotional openness helps to build stronger teams

***Jordi L Tremoleda***

Working with laboratory animals remains challenging. Researchers are faced with a complex working environment where they work to a highly-valued regulatory framework; whilst, at the same time, they are challenged by the expectations to deliver successful and impactful scientific discoveries. Furthermore, research and technical staff are committed to delivering the best animal care and welfare support even though they recognise that such experimental interventions will intrinsically cause some pain, suffering or distress to the animals. Undoubtedly, confronting these scenarios directly impacts on the emotional bond that staff develop towards the animals under their care. These emotional and professional relationships that researchers manage with their research animals are finely balanced by the support from personally-committed laboratory animal technicians. These technicians are recognised for the importance of their work in protecting the welfare of the animals. This fine balance remains critical and must be protected and recognised; along with all the regulations and animal care guidelines, as it is the staff’s personal commitment to the animal’s wellbeing which really makes a clear caring impact on the animals. Unfortunately, the research culture is mostly described as a competitive, pressured, insecure, challenging, unsupported and stressful environment as outlined by a recent survey ( Wellcome Trust, 2020, p. 202). Such an image hinders a healthy environment that can develop a caring, compassionate, respectful and affective culture of care for the animals and importantly, for the staff to also feel appreciated, cared-for and supported: these values are often referred to as ‘a culture of care’.

Following the Brown report (2014) energy to support and define what a ‘culture of care’ means and could achieve in the animal research industry began to grow ( Boden & Hawkins, 2016). The aim of the culture of care is to promote a more supportive and empathetic environment at work, and to acknowledge the emotional challenges associated with our work with animals. It is important to create physical and emotional spaces where staff can feel comfortable and empowered to communicate, to facilitate cross-cultural and disciplinary discussion engaging with all the different staff in the teams, and to embrace “a team sharing responsibility approach”. Such collective, yet personally driven approaches, are key to ensuring a respectful, inclusive, and compassionate communication approach, but how can they be orchestrated? In this discussion, the use, and reasons for, experimenting with a new storytelling tool, developed to facilitate emotional openness is reflected upon. The workshop addresses the need for a space to address emotional challenges in animal professionals’ work. The “Care-full Stories” tool uses fictionalised prompts (storytelling) to encourage participants to share their own stories from working in animal research, through facilitating in-depth, open, conversations. This short article synthesises the experiences of author Tremoleda, co-leading research engagement workshops with animal research industry professionals about their experiences; these were not research data collection exercises. Consequently, no application for institutional ethical approval took place (see Garcia-Iglesias *et al.*, 2024; Nollett *et al.*, 2024) and participation was voluntary and not systematic.

This recent use of the Care-full Stories tool was part of a wider animal research community response to loudly recognise the importance of emotion and feelings in their work. Emotion and feeling are used as an instrument by animal professionals to drive forward the best care and welfare of lab animals, and the best care to staff and colleagues. A greater awareness for the emotional relevance of this aspect to their work has rapidly evolved. In the last two years, there have been a number of initiatives ( Young *et al.*, 2024) ( Ferrara *et al.*, 2022) to speak more of the emotionally complex working environment of laboratory animal research, that have come under the umbrella phrase, to nurture a ‘culture of care’ ( Tremoleda & Kerton, 2020). These have been translated into the production of various guidance and supportive documents ( Tremoleda *et al.*, 2023). Such supportive tools have led to great improvements in the culture of care and better ways to support staff, and these played a crucial role during the Covid-19 Pandemic. Animal research communities were drastically impacted by the pandemic, triggering a desire to properly acknowledge, value and engage with the emotional labour of animal professionals. In a very short time-frame, hard, unprecedented decisions had to be taken to manage animal units during the lockdown periods, enforcing emergency decisions involving the emergency killing of a considerable number of animals to reduce animal stock, stopping studies that were running, changes to working conditions, not being exempt to personal health risks, or simply the heart-breaking decisions to immediately close units. While nobody could predict the impact of the COVID outbreak, all these emergency actions took a great toll on the lab animal community, building up incredible emotional pressures on staff. Many from this community, shared the feeling a tipping-point was reached that overwhelmed the emotionally complex, tight-balance of their work. Despite the commitment and emotional resilience of the community, the drastic action within the pandemic, led to a remarkable emotional dissonance and frustration in staff. This clearly exposed the fragility of the emotional balance associated with laboratory animal work.

Using the “Care-full Stories” tool facilitated emotionally open discussions, that addressed the lack of space to address emotional challenges in their work. The “Care-full Stories’ tool uses fictionalised prompts (storytelling) to encourage participants to share their own stories from working in animal research, facilitating in-depth conversations and openness ( Greenhough & Mazhary, 2021). The activity aims to build emotional connections within the professional duties of other professionals/researchers of the team by the sharing of personal experiences. It works to build appreciation that there are different kinds and understandings of care within the team, through engaging participants in discussions on different attitudes and challenges in the lab animal working environment. The tool has been designed and piloted in the UK, as part of the Wellcome-funded Animal Research Nexus Project. The storytelling approach was selected as a great opportunity for participants to draw on their own experience at work and narrate their own stories as a way of sharing particularly emotionally or ethically challenging situations that they have experienced. The tool provides a set of fictionalised scenarios that have to be represented by voluntary members of the discussion group, by means of simply reading the script or engaging with more theatricality. To ensure a good state of mind at the start of the workshop, a facilitator will provide an overview of the activity, assuring the importance of establishing a ‘safe and confidential space’. To this aim, a short ice-breaking activity is carried out to create a relaxed and friendly tone for the workshop. This is then followed by a script reading of a selected scenario; the tool provides various possible scenarios that can be chosen based on the specific of the facility and team expertise. Facilitators are encouraged to ask for volunteer ‘readers ‘for the script; during the pilot activities it was found that the activity worked best when volunteers read a role different from their own. Once the story has been read / performed, to facilitate open reflection and sharing of opinions, the tool provides a set of recommended questions. These are to provide guidance but can be easily adapted based on the group dynamics to allow the conversation to flow and explore topics beyond those suggested. At the end of the session participants are encouraged to reflect on what they had learned, sharing it through a group open discussion. As a nice reminder of the event, and to reinforce future commitments, the training resource contains useful examples of a “pledge postcard”, which participants can complete at the end of the session and facilitators arrange to post back to them a few months later. This is intended to be a useful prompt or aide memoire to put learning into action once participants have returned to the workplace.

In this use of the Care-full Stories tool, participants commented positively on the interactive nature of the storytelling approach, playing different roles, and seeing everyday workplace challenges from different perspectives within the team. It was observed how successfully it supported good engagement amongst participants. The team role-playing discussions seemed to trigger individuals to openly talk about their own personal, professional, and emotional challenges. And these challenges were met by those present with expressions of empathy. These conversations have triggered greater reflections for a need to be more empathetic and supportive as a working team. Feedback from the activity clearly indicated the value placed on open communication, and the need to promote safe and open environments that allow different staff to speak openly, and to feel supported and cared for independently of their status and responsibilities. Since the session on ‘Creative methods in animal research’ in March 2023, we have carried out various events using the “Care-full Stories” tool in various professional setting including pharma, biotech and academic settings across various countries in Europe and South America. The feedback and engagement have been extraordinary, highlighting the urge to facilitate and importantly, acknowledge the emotional aspects of our work. Thus, it is through how this activity supports the exploration of ‘others’ roles and responsibilities, to acknowledge the challenges faced by different professionals and to understand the important role of how we communicate this in our daily work, that is a legacy of working with the tool. In the long term we hope it can support building better bridges for emotional communication that can help to openly recognise the valuable work of the laboratory animal professionals and their commitment to ensure the best care of the animals.

## Concluding discussion

Bella Lear ( Roe *et al.*, 2024a), from Understanding Animal Research, describes how the animal research community has moved ‘from one of an uncertain and concerned sector, to a recognition that communication with those beyond their sector is not only possible, but desirable’ (p. 412). In this article the opportunities around creative embodied methods are discussed for breadthening tactics surrounding that communication about animal research with those outside the sector, and those working within it. In this conclusion there is a review of the different creative skills, opportunities and applications identified across the examples in this paper, to make the case that the guidance and vision of communication about animal research, articulated in the Concordat, could be revised to value and foster these creative contributions. This includes loosening control about the message; to foster opportunities to create spaces for becoming curious, as opposed to fearful, about animal research; to share experiences about, and from, the research animal community; to be confident about presenting the humanity, not the ‘Frankenstein’, that centres animal industry practices. Importantly, these actions are as relevant for those working with animals inside the industry as those outside them.

The three examples of creative methods in practice featured in this paper demonstrate what opportunities arise through experiences that allow participants to feel the pull of their own curiosity to renew and revise associations, feelings, meanings related to animal research. In the first example (Mackenzie) demonstrates the value of engaging arts professionals early in conversations around performing animal research for public audiences. The latter two examples (Bailey and Tremoleda) evidence how AnNex experiments with openness, particularly around the contribution creative methods can make, are now being taken up by people beyond the AnNex team. They illustrate how to engage participants working with research animals, and those not, through providing a creative experience of learning through embodied practices – creating scenarios that identify with the position of the experimental subject, sewing and connecting to the life of a research mouse taking shape in your hands, or listening and learning through role-playing scripted conversations: these experiences disrupt traditional rational thought-based knowledge exchange. Instead, these embodied experiences operate as creative acts for learning and relearning, opening space for new meanings and feelings towards animal research.

Creative embodied methods unsettle didactic processes of learning between an expert and the learner. Formal scientific methods and materials for communicating are set aside and replaced with practical, experiential interventions. These creative methods allow animal research to be a topic for generating self-understanding, self-reflection, new knowledges, on hard to talk about subjects; this is achieved alongside a commitment on one hand to playful and creative making, whilst on the other hand, foregrounding conversations on complexity and contradiction. In these examples, creative embodied methods do more than gather academic research data (cf von Benzon *et al.*, 2021). Creative embodied methods can be employed for the purposes of participatory engagement in social science research (not always by artists) and in creative approaches in art research which are intended to engage multiple audiences on many levels, one of which may be a (social science) research theme. In this paper this is illustrated through themes around animal research, care and experimentation. The three reflections from an artist, a vet and a pharmacologist engaging creative embodied methods for artistic performance and participatory activities related to animal research, illustrate how they are a vehicle for successful classroom teaching, public and professional engagement experiences, on this sensitive topic. What links the examples are a foregrounding of embodied experiences, forged from taking part or experiencing an activity that requires learning and sharing expertise through doing, as opposed to receiving learning by reading or listening to talk, illustrated across Mackenzie’s performative art practice, Bailey’s experiences of letting her first-year pharmacology students sew laboratory mice and Tremoleda’s explorations of caring through scripted conversation. These are each transformative lived experiences for those involved because they prioritize accessing an individual’s active creativity and expertise, whether artist, undergraduate student or animal technologist, over delivery of tailored public relations’ communications that rely on passive open-eared recipients. These methods foster staying with emotion, uncertainty, complexity and confusion as opposed to settled rational thought and argument.

Notably, the content of each activity featured is not centered on making ethical argument and debate but rather learning of the world and how one can find a way to relate to it through a personalising approach. Whether making and holding a felt mouse and then not being able to take it home, to role-playing conversations that speak to the challenges of animal caring, to installations, workshops or films where artists engage with animals to ethically perform aspects of animal research and animal welfare (outside of the normal conventions of scientific experimentation), - each bursts with fresh articulations and angles to deepen and broaden how animal research is known, contributing to the field where creatives Maisie Tomlinson and the Lab Collective and others also work. The methods offer a way to share the humanity of relating to animals in its complexity, found in those who work in the industry and wider society, as opposed to an internet search for data about it. They address the question what happens when, rather than how, or if, people choose to engage with animal research ( Roe *et al.*, 2024a)? As in each case what performing animal research provides is a way into the experiences of people working in animal research and the relations they make with research animals, as opposed to public facing information on animal health and welfare and non-technical summaries about the research. These approaches pass the reins to participants. This is an approach that is of its time, as we find ourselves in an era where traditional forms of expertise and knowledge gate-keeping have been dismantled with the rise of Social Media and confusion around ‘facts’. There is no prioritizing of the ‘facts’ in these engagements with animal research, indeed there is the active use of fiction to explore terrains that can’t be known or encountered first-hand. As such there is an underlying acceptance that the participant brings skills and can develop them through the activity, about how to handle and sort through different types of information and form their own position.

This exploration into self-guided embodied creative practices in animal research highlights the need to revisit the Concordat on Openness in Animal Research, over a decade since its inception. Creative arts are opening new pathways for engagement both within and beyond the industry. While the Concordat is designed for external communication, this paper shows its internal impact - , enhancing wellbeing, job satisfaction, and staff retention among researchers and technicians. Initiatives like Care-full Stories and The Mouse Exchange are being used to foster a workplace culture of care, supporting reflection and dialogue about caring for both animals and staff. The Concordat could be revised to recognize the value of openness within the industry, not just externally. Creative methods offer powerful tools for industry professionals to explore their experiences and bring change to the style of public engagement on animal research. They also encourage curiosity in the broader biomedical industry, balancing ethical use of animals where there is a regulatory or scientific need, with innovation in alternative non-animal methods. Interdisciplinary perspectives, especially from non-STEM fields, can foster an evolution in public engagement formats from one-way communication to inclusive dialogue—inviting both publics and professionals to shape the conversation. In recognition of the contributions detailed in this paper, the Concordat could emphasize diversification in the type of disciplines that have a role to play in how the Openness agenda is achieved.

## Ethics and consent

This paper does not report on a research study of humans. Instead, it includes reflective accounts of education activities run by Bailey and workshops run by Tremoleda, and as such thus there is no experimental data collected or analysed. Consequently an application for institutional ethical approval to run these events was not required, and participant consent was given by choosing to attend the activities that were opportunities to learn. In the case of Bailey - delivering the Mouse Exchange as a student-led education activity - the activity is a teaching intervention informed by research and may inform research, but is not in itself research. Tremoleda’s experience of trialing and delivering the Care-Full stories workshops with animal research professionals in various settings, documents personal and collective experiences and feedback; again, this is not research. No animals were used in these studies, and participants engaging with the trial attended at their own discretion to learn more about, and from, these initiatives. No personal data is presented from the individual attendees, nor has human research data been used or presented in this reflective essay.

## Data Availability

The Mouse Exchange engagement toolkit, discussed by Bailey is available at http://eprints.soton.ac.uk/id/eprint/453157. Carefull-Stories interactive training resources, as discussed in use by Tremoleda, are available at https://www.geog.ox.ac.uk/research/technological-life/projects/care-full-stories/index.html. The Mouse Exchange toolkit is distributed under Creative Commons License – CC BY-NC-SA. Carefull-Stories training sources are distributed under a CC-BY license. Works of art that Mackenzie has created and discusses can be learnt more about at https://www.loumackenzie.com. The classroom-based outcomes discussed by Bailey, which did not require institutional ethical review, is not suitable as a share-able dataset. Please contact Bailey at S.Bailey@bath.ac.uk to discuss access. However, www.themouseexchange.org website, under the Colonies tab, does contain a share-able image-bank of outputs from historic Mouse Exchange events.
