# Supplementary material for: Creative pedagogic methods for self-guided embodied thinking and learning about animal research
Source: Wellcome Open Res. 2025 Oct 24;10:595. [Version 1] doi: 10.12688/wellcomeopenres.24930.1 (PMC13032104; doi:10.12688/wellcomeopenres.24930.1)
Supplement: Peer review history

# Reviewer report: Reviewer response for version 1

**DOI:** 10.21956/wellcomeopenres.27460.r148817

**Published:** 2026-03-31

**Authors:** Daniel M. Weary

**Affiliations:** 1 The University of British Columbia, Vancouver, British Columbia, Canada

Thanks for this very interesting and helpful paper. I think this could be an important contribution, but the current paper is not written in the format of a traditional ‘research article’ which means that the authors will need to do extra work to explain what they are doing, why this is important, why the contributions are novel, and how exactly their contributions moves the field forward (as well as any limitations and areas for future work). 

 To begin with, the authors need to clarify exactly what this paper is supposed to be; they take pains to explain that this is not a research contribution, yet it is submitted under the category of ‘research article’. They are also say it is not a methods paper, even though the main contribution seems to be in describing 3 different types of intervention. My feeling is that this is a type of methods paper (just not strictly a research methods paper). Following this logic, I’d like to see more detail (and a more consistent level of detail) in terms of exactly what each method consists of, as well as the relative benefits and weaknesses of these approaches.

 Another challenge is that the three substantive sub-sections (describing the different methods), is each penned by a different author. This format means that the voice differs across the three sections, which is not in itself a problem but does mean that reading is a little more work. More importantly, the substance of the three sections varies considerably. The first is introspective, the second is data rich, and third is a more simple description; given the theme of the paper I can see some benefit of letting the authors tell their own stories, but without more context this makes it hard for the reader to know where to focus.

 In the Discussion, I’m left with the impression that much of what is said could have been written in absence of the three examples. I’d like to see more about how this examination of the three methods moves the field forward. It would also be useful to walk the reader through how these methods meet (or fail to meet) the broader objectives outlined in the introduction. I’d also like to see some discussion of other methods that could be applied (perhaps in a Table), together with some discussion of how various methods should be used or even combined depending upon pedagogical aims.

Is the work clearly and accurately presented and does it cite the current literature?

Yes

If applicable, is the statistical analysis and its interpretation appropriate?

Not applicable

Are all the source data underlying the results available to ensure full reproducibility?

No source data required

Is the study design appropriate and is the work technically sound?

Yes

Are the conclusions drawn adequately supported by the results?

Partly

Are sufficient details of methods and analysis provided to allow replication by others?

No

Reviewer Expertise:

Animal welfare and the ethics of animal use

I confirm that I have read this submission and believe that I have an appropriate level of expertise to confirm that it is of an acceptable scientific standard, however I have significant reservations, as outlined above.

# Reviewer report: Reviewer response for version 1

**DOI:** 10.21956/wellcomeopenres.27460.r148811

**Published:** 2026-03-27

**Authors:** David Mawufemor Azilagbetor

**Affiliations:** 1 University of Basel, Basel, Switzerland

**Summary of the paper**

 This paper offers a timely contribution to the important area of public communication about animal research. While general communication efforts often focus on what researchers and institutions believe the public should know, this paper discusses the importance of creating space, through creative approaches, for publics to reflect more broadly on animal research. The overarching creative methods presented include designing labels for medicines that indicate the use of animals in their development; creating a synthetic, fictional laboratory mouse to facilitate open dialogue about laboratory animals; engaging participants in harm-benefit analysis through a game simulation in which they assume the role of an ethical review body member; and creating spaces for open discussion about the psychological burden borne by animal research personnel. These creative, self-guided approaches to thinking and learning about animal research provide opportunities for intended audiences to engage with its complexities, rather than being limited to the information that communicators choose to provide.

**Major comments**

***References***

 In general, the manuscript would benefit from additional citations to support the various arguments and to better ground its rationale. In particular, references are needed in the following cases:

- Many of the arguments presented in the third paragraph of the Introduction, concerning the need to account for the complexity of public sentiment in communication about animal research, require further supporting references. At present, only one source (Law, 2004) is cited at the end of the paragraph.
- On page 12, references are needed to support the discussion of the mass culling of laboratory animals during the COVID-19 pandemic

***Intended publics for these creative approaches***

 The paper is generally framed within the context of public communication about animal research. In the Introduction, the authors reflect “upon the intended audience for these communications,” with particular emphasis on the general public. However, a substantial portion of the creative methods primarily involved internal stakeholders in animal research, including life sciences students and animal research professionals, who may already have closer engagement with animal research than the general public. The conclusion of the paper specifically recommends that the Concordat “could be revised to recognize the value of openness within the industry, not just externally”. I think these creative approaches have huge potential in discussing animal research with the general public as well.

 The authors could provide a clearer indication and framing that the creative methods were largely intended for internal audiences. But more preferably, the authors could offer further reflection and discussion on extending these creative engagement approaches to external stakeholders, particularly the general public, and the potential outcomes (similar to or different from those for internal publics) within the general public. This would enable a stronger consideration of self-guided thinking and learning about animal research among external audiences such as the general public, as the paper is currently framed to achieve.

***How these creative methods lead to discussions about the complexity of animal research***

 One of the main rationales of the paper is to create space for participants to explore the complexities, often around ethical trade-offs, surrounding animal research. The authors could reflect a bit on how these creative methods reveal the complexity of animal research and enable participants to engage in discussion and reflection on the various dilemmas that may arise, in particular, how the broader discussions relate to the (im)permissibility of animal research.

**Minor comments:**

 1. Remove the “s” from “raises” in the sentence: “Observations of these examples raises ……”

 2. In Box 1: Add “i” to “mce” in the sentence: “How mce are treated during an experiment”

 3. Page 13: Is (are) there any word(s) missing from this sentence “the tool provides various possible scenarios that can be chosen based on the specific of the facility and team expertise.”?

Is the work clearly and accurately presented and does it cite the current literature?

Partly

If applicable, is the statistical analysis and its interpretation appropriate?

Not applicable

Are all the source data underlying the results available to ensure full reproducibility?

No source data required

Is the study design appropriate and is the work technically sound?

Yes

Are the conclusions drawn adequately supported by the results?

Yes

Are sufficient details of methods and analysis provided to allow replication by others?

Yes

Reviewer Expertise:

Animal Research Ethics; Harm-Benefit Analysis; Patient and Public Involvement in Animal Research

I confirm that I have read this submission and believe that I have an appropriate level of expertise to confirm that it is of an acceptable scientific standard.

# Reviewer report: Reviewer response for version 1

**DOI:** 10.21956/wellcomeopenres.27460.r137422

**Published:** 2025-11-24

**Authors:** Megan Lafollete

**Affiliations:** 1 The 3Rs Collaborative, Denver, USA

Excellent paper that addresses an important, timely, and understudied area that lacks relevant publications. Many excellent recommendations and thoughtful commentary on innovative ways to engage the public related to animal research. I appreciate the practical case studies and interdisciplinary approach.

 That being said there are a few areas of improvements.

 General recommendations throughout

- Define terms like “embodied learning” “affective realism” and “somatic sensibilities” and consider less “technical” terms. As the manuscript is written, some of its target audience (e.g., scientists working in animal research) may struggle with understanding these concepts
- The paragraph structure is very lengthy with a number of paragraphs taking up an entire column on a page. Again, this may lose some of the desired readership. I recommend rephrasing into shorter paragraphs with clear topic sentences with supporting evidence to enhance reader comprehension.

Introduction:

- I think that it’s an oversimplification that the thought by the industry is just about how people who respond differently to public surveys about animal research. Rather I think the hope is that if people were better informed and understand animal research that they would genuinely be more supportive and accepting of animal research, which in turn would be reflected by public opinion surveys. Right now the first paragraph kind of make it seems like that’s what the field cares about versus making real change.
- In reviewing current efforts, there is a lack of noting of efforts such as Biomedical Research Awareness Day (BRAD) or Be Open about Animal Research day (BOARD) which typically include in person events that allow for two-way conversations: https://www.bradglobal.org/how-to-participate

Common formats include:

  

- Tabling Events: Share information and engage in conversations with passers-by while distributing educational materials and giveaway items.
- Classroom Takeovers: Bring BRAD to any age and classroom size to incorporate animal research into an educational setting. 
- Community Talks or Lunch & Learns: These are ideal for educating local groups about the role of animal research in advancing health and science.
- Seminars or Webinars: Virtual events that expand your reach and allow you to host experts discussing topics related to animal research.
- Science or Trivia Nights: Make science fun by testing participants knowledge in an engaging and more casual setting. 
- Interactive activities: Include games, jeopardy, a scavenger hunt and more in your celebration to add some friendly competition.
- Tour of research facilities and/or vivarium: Show off the incredible care animals in research receive. 

-Page 11 and Page 12: most modern & refined definitions of a culture of care includes focus on animal welfare, care of staff, openness, and scientific quality. So at the end of page 11 please change to “these values are a key part of a “culture of care”. Otherwise it over simplifies the concept and misses out on two key pillars. The next paragraph continues to over-simplify the concept and confuse it with a primary focus on staff well-being, which is important, but only one of the pillars. This paragraph should be rephrased to include more modern definitions. See this publication: https://pmc.ncbi.nlm.nih.gov/articles/PMC7564339/

- The paper suggests revising the Concordat but does not provide concrete recommendations. Add specific wording or principles that could be included in an updated Concordat.
- The claim that the UK government has committed to phasing out animal research may be overstated. Current policy supports reduction and refinement, but full phase-out is not official. Recommend rephrasing to “long-term goal to reduce reliance on animal research and accelerate non-animal methods.”

Is the work clearly and accurately presented and does it cite the current literature?

Yes

If applicable, is the statistical analysis and its interpretation appropriate?

Not applicable

Are all the source data underlying the results available to ensure full reproducibility?

Yes

Is the study design appropriate and is the work technically sound?

Yes

Are the conclusions drawn adequately supported by the results?

Yes

Are sufficient details of methods and analysis provided to allow replication by others?

Yes

Reviewer Expertise:

Human behavior change, science communication, 3Rs, animal research,

I confirm that I have read this submission and believe that I have an appropriate level of expertise to confirm that it is of an acceptable scientific standard.
